# Achieving a cure for anastomotic leakage following laparoscopic low anterior resection for rectal cancer using an endoscopic closure device, a MANTIS clip

**DOI:** 10.1093/jscr/rjae523

**Published:** 2024-08-25

**Authors:** Daisuke Horikawa, Hiroki Takahata, Yasuhiro Fujiwara

**Affiliations:** Department of Surgery, Furano Kyokai Hospital, Furano, Hokkaido 076-8765, Japan; Department of Surgery, Furano Kyokai Hospital, Furano, Hokkaido 076-8765, Japan; Department of Surgery, Furano Kyokai Hospital, Furano, Hokkaido 076-8765, Japan

**Keywords:** anastomotic leakage, rectal cancer, low anterior resection, MANTIS clip, endoscopic clip, endoclip

## Abstract

Anastomotic leakage (AL) following low anterior resection (LAR) for rectal cancer is a major complication. While most reports focus on the closure of AL using over-the-scope clip (OTSC), few reports are available on the use of through-the-scope clip (TTSC). This is because TTSC is not typically designed for full-thickness closure, unlike OTSC. However, a MANTIS clip, categorized as TTSC, is indicated for full-thickness closure. A 73-year-old man diagnosed with AL 7 days postoperatively following laparoscopic LAR underwent laparoscopic drainage and ileostomy the next day. Although the drainage led to the shrinkage of the fistula, it persisted even after 2 months. Consequently, the fistula orifice was closed using a MANTIS clip under colonoscopy and radiography. Two days later, the patient was discharged. The drain was withdrawn cautiously to prevent residual fistula and removed completely on day 29. This report highlights our experience in using a MANTIS clip for AL following LAR.

## Introduction

**Figure 1:**
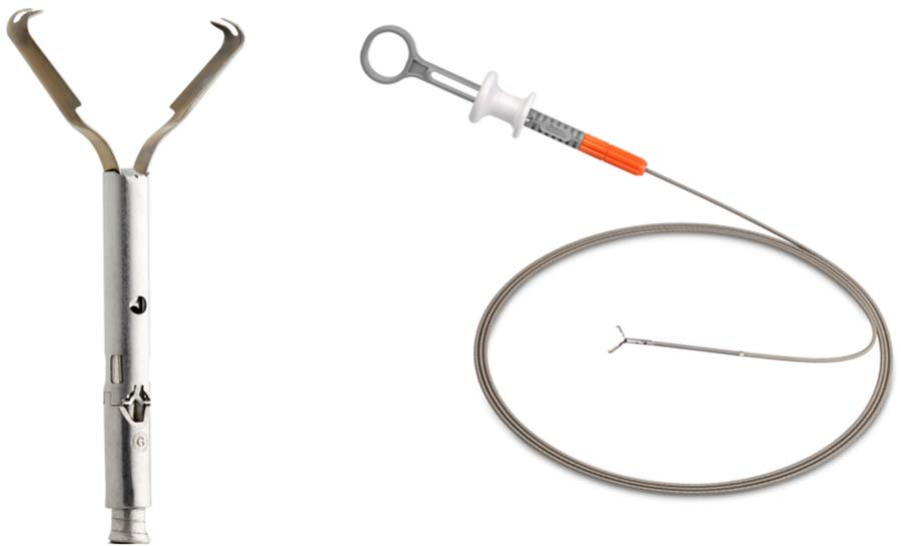
MANTIS closure device (Boston Scientific Corporation, Marlborough, MA, USA)

Anastomotic leakage (AL) is a major complication after laparoscopic low anterior resection (LAR) for rectal cancer. The role of endoscopic clips in achieving AL and fistula closure remains unestablished. Only a few reports are available on using through-the-scope clip (TTSC) for AL closure after LAR. Herein, we present a case involving the use of a MANTIS clip, a TTSC (Fig. 1), for AL closure after laparoscopic LAR for rectal cancer.

## Case report

A 73-year-old man was referred to our hospital following a positive fecal occult blood test result. Total colonoscopy and biopsy revealed a carcinoma of 15 mm in diameter in the lower rectum. Because of the tumor’s invasion in the deep submucosal layer, as shown on endoscopic ultrasonography, laparoscopic LAR with D3 dissection (Japanese Society for Cancer of the Colon and Rectum [JSCCR] 9^th^ edition) using the double stapling technique and a 25-mm transanal circular stapler was performed. A drain was placed in the pelvis during the procedure that lasted for 281 minutes with minimal blood loss. The tumor was staged as T1b, N0, M0, Stage I based on the JSCCR (9th edition) and T1, N0, M0, Stage I based on the Union for International Cancer Control (8th edition) criteria.

The patient commenced oral feeding 4 days postoperatively. The next morning, a serous discharge was observed at the drain; hence, the drain was removed. Later that evening, the patient developed a high fever without other symptoms. His white blood cell (WBC) count was 9820 /μL, and C-reactive protein (CRP) level was 5.09 mg/dL. Computed tomography (CT) revealed only air along the drain placement site. However, an AL was suspected due to fever, and fasting was implemented. The patient’s condition remained stable for 2 days, but the abdomen gradually distended. The WBC count increased to 11 900/μL and CRP level elevated to 18.59 mg/dL. Rectal contrast imaging confirmed leakage on the right wall of the anastomosis. Additionally, CT showed leakage of contrast medium out of the anastomosis and an abscess in front of the sacral promontory. On postoperative day 8, laparoscopic drainage and ileostomy were performed.

**Figure 2:**
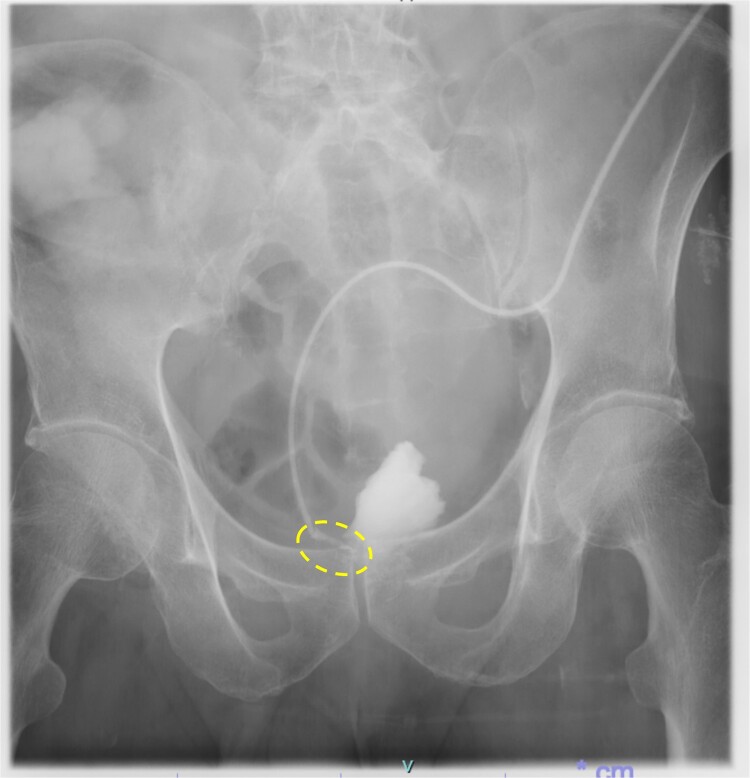
Persistence of fistula even after 2 months of re-operation (dotted circle)

**Figure 3:**
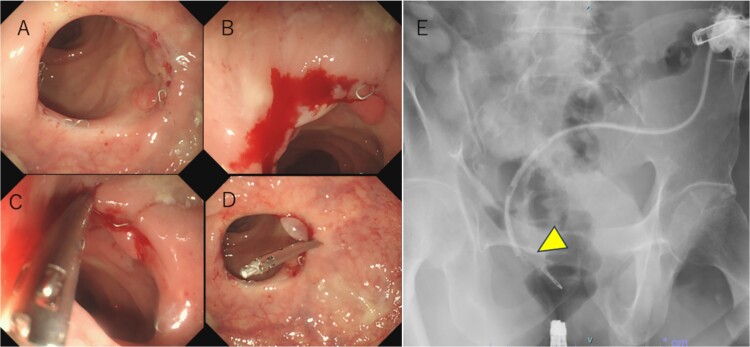
(a) Hyperplastic granulation tissue around the right side of the anastomosis. (b) Minor bleeding is observed after the gastrografin is injected through the drain. (c) A suspected fistula orifice site is closed provisionally using a MANTIS clip. (d, e) After provisional closure of the fistula orifice with a MANTIS clip, gastrografin is injected again through the drain, and no anastomotic leakage is observed (arrowhead). Subsequently, a definitive clipping is performed.

**Figure 4:**
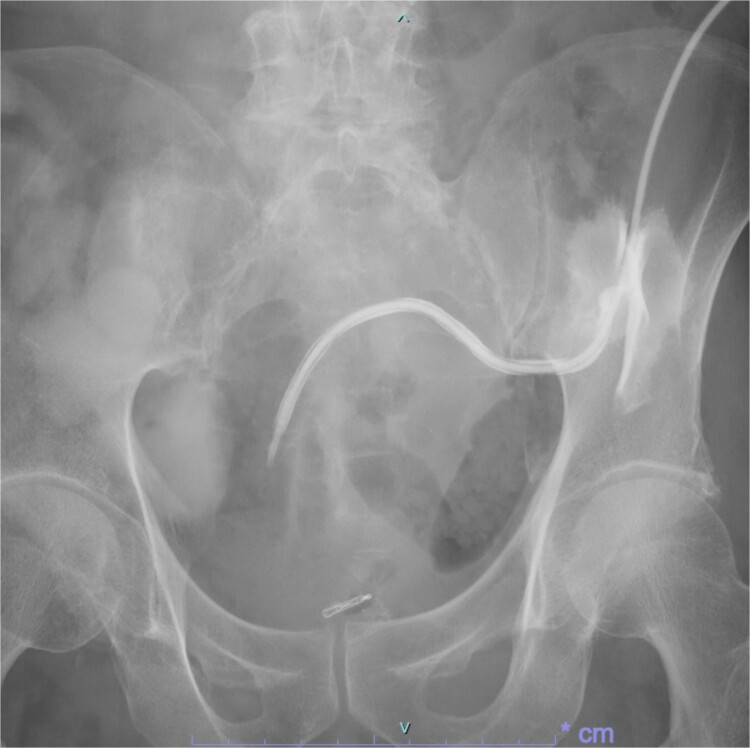
On day 15 after clipping, gastrografin injection under radiography reveals the fistula tends to recover.

Following reoperation, although drainage led to shrinkage of the fistula, it persisted after 2 months (Fig. 2). Hence, we decided to close the fistula using endoscopic clips. Gastrografin was injected through the drain under colonoscopy and radiography, and bleeding was observed at the suspected fistula orifice, which was provisionally clipped using a MANTIS clip. Gastrografin was injected again, and definitive clipping was completed confirming the successful fistula closure (Fig. 3). The patient was discharged after 2 days. The drain was withdrawn about 3 cm on day 9 after discharge. On day 15, gastrografin injection under radiography revealed recovery of the fistula (Fig. 4). Thereafter, the drain was withdrawn cautiously about 2 cm every 2–3 days to prevent residual fistula formation and removed completely on day 29. Ileostomy closure was performed 6 months after the initial surgery. The patient was discharged without complications on postoperative day 7.

## Discussion

AL, a critical complication following rectal surgery, occurs in approximately 10–13% of patients [1, 2] and results in increased costs, duration of hospitalization, rate of local recurrence, and cancer-specific mortality [3, 4].

**Figure 5:**
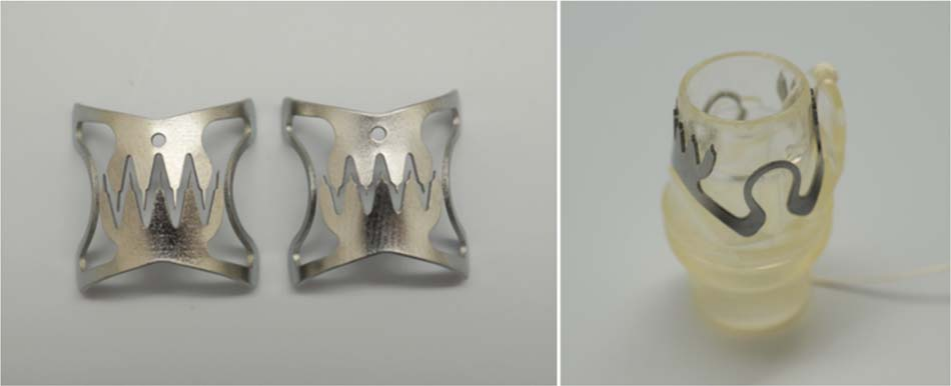
Over-the-scope clip (Ovesco Endoscopy AG, Tubingen, Germany)

Some endoscopic management techniques of AL include vacuum-assisted closure therapy, using fibrin glue, stent, and endoclips [5]. Endoclips are of two types: TTSC and over-the-scope clip (OTSC) (Fig. 5).

OTSC is mounted on a cap installed at the tip of the endoscope, which allows full-thickness closure up to 2 cm [5]. However, it requires a complex setup before clipping and has limited repositioning capabilities [5]. Kobara et al. [6] reviewed 1517 cases of AL closure using OTSC, reporting clinical success rates of 84.6, 51.5, and 66% for perforation, fistula, and AL, respectively. Although fistula has the lowest success rate, clinical success can be achieved only for relatively small fistulas (<10 mm) using a single OTSC. Negm et al. [7] reported that all 24 cases of fistula or AL after LAR for rectal cancer completely recovered using OTSC; the indication for OTSC usage in their study was fistulas of <10 mm.

TTSC is a readily available accessory that can be inserted through the operative channel of the scope [5]. However, it is not typically used for full-thickness closure. Only few case reports on the management of AL after LAR for rectal cancer with TTSC are available [8, 9]. To our knowledge, this is the first documented case report of managing AL using a MANTIS clip.

The MANTIS clip, categorized as TTSC, is easy to use because of its similarity with the conventional TTSC. It is indicated for full-thickness closure up to 2 cm and offers several advantages. First, it is less expensive, with the cost of one MANTIS clip being approximately one-quarter that of one OTSC. Second, it does not require complicated deployment procedures before clipping. Finally, the most important feature is that a MANTIS clip can be re-clipped and re-positioned, thus eliminating clipping errors. Although a MANTIS clip is indicated for full-thickness closure up to 20 mm, it may also be effective in cases of fistula or AL <10 mm, given the high effectiveness of OTSC in these situations.

In previous reports [8, 9], where the drain had not been removed when AL was suspected, the AL was successfully managed using only endoclips. However, in our case, the drain had been removed when AL was suspected. Had the drain remained, the AL could have been managed using endoclips alone. Moreover, because the AL was localized and the drainage was adequate, earlier closure of the fistula may have been achieved using endoclips.

Despite the best efforts, surgeons cannot eliminate AL after LAR for rectal cancer, and some patients develop refractory fistula even with appropriate drainage. A MANTIS clip could be effective in cases of fistula or AL after LAR for rectal cancer.
